# Where No Universal Health Care Identifier Exists: Comparison and Determination of the Utility of Score-Based Persons Matching Algorithms Using Demographic Data

**DOI:** 10.2196/10436

**Published:** 2018-12-13

**Authors:** Anthony Waruru, Agnes Natukunda, Lilly M Nyagah, Timothy A Kellogg, Emily Zielinski-Gutierrez, Wanjiru Waruiru, Kenneth Masamaro, Richelle Harklerode, Jacob Odhiambo, Eric-Jan Manders, Peter W Young

**Affiliations:** 1 Division of Global HIV and TB Centers for Disease Control and Prevention Nairobi Kenya; 2 Global Programs for Research and Training University of California San Francisco San Francisco, CA United States; 3 National AIDS and STI Control Program Ministry of Health Nairobi Kenya; 4 Institute for Global Health Sciences University of California San Francisco San Francisco, CA United States; 5 The Palladium Group Nairobi Kenya; 6 Division of Global HIV and TB Centers for Disease Control and Prevention Atlanta, GA United States

**Keywords:** deterministic matching, score-based matching, HIV case-based surveillance, unique case identification, universal health care identifier

## Abstract

**Background:**

A universal health care identifier (UHID) facilitates the development of longitudinal medical records in health care settings where follow up and tracking of persons across health care sectors are needed. HIV case-based surveillance (CBS) entails longitudinal follow up of HIV cases from diagnosis, linkage to care and treatment, and is recommended for second generation HIV surveillance. In the absence of a UHID, records matching, linking, and deduplication may be done using score-based persons matching algorithms. We present a stepwise process of score-based persons matching algorithms based on demographic data to improve HIV CBS and other longitudinal data systems.

**Objective:**

The aim of this study is to compare deterministic and score-based persons matching algorithms in records linkage and matching using demographic data in settings without a UHID.

**Methods:**

We used HIV CBS pilot data from 124 facilities in 2 high HIV-burden counties (Siaya and Kisumu) in western Kenya. For efficient processing, data were grouped into 3 scenarios within (1) HIV testing services (HTS), (2) HTS-care, and (3) within care. In deterministic matching, we directly compared identifiers and pseudo-identifiers from medical records to determine matches. We used R stringdist package for Jaro, Jaro-Winkler score-based matching and Levenshtein, and Damerau-Levenshtein string edit distance calculation methods. For the Jaro-Winkler method, we used a penalty (р)=0.1 and applied 4 weights (ω) to Levenshtein and Damerau-Levenshtein: deletion ω=0.8, insertion ω=0.8, substitutions ω=1, and transposition ω=0.5.

**Results:**

We abstracted 12,157 cases of which 4073/12,157 (33.5%) were from HTS, 1091/12,157 (9.0%) from HTS-care, and 6993/12,157 (57.5%) within care. Using the deterministic process 435/12,157 (3.6%) duplicate records were identified, yielding 96.4% (11,722/12,157) unique cases. Overall, of the score-based methods, Jaro-Winkler yielded the most duplicate records (686/12,157, 5.6%) while Jaro yielded the least duplicates (546/12,157, 4.5%), and Levenshtein and Damerau-Levenshtein yielded 4.6% (563/12,157) duplicates. Specifically, duplicate records yielded by method were: (1) Jaro 5.7% (234/4073) within HTS, 0.4% (4/1091) in HTS-care, and 4.4% (308/6993) within care, (2) Jaro-Winkler 7.4% (302/4073) within HTS, 0.5% (6/1091) in HTS-care, and 5.4% (378/6993) within care, (3) Levenshtein 6.4% (262/4073) within HTS, 0.4% (4/1091) in HTS-care, and 4.2% (297/6993) within care, and (4) Damerau-Levenshtein 6.4% (262/4073) within HTS, 0.4% (4/1091) in HTS-care, and 4.2% (297/6993) within care.

**Conclusions:**

Without deduplication, over reporting occurs across the care and treatment cascade. Jaro-Winkler score-based matching performed the best in identifying matches. A pragmatic estimate of duplicates in health care settings can provide a corrective factor for modeled estimates, for targeting and program planning. We propose that even without a UHID, standard national deduplication and persons-matching algorithm that utilizes demographic data would improve accuracy in monitoring HIV care clinical cascades.

## Introduction

In Sub-Saharan Africa, HIV case-based surveillance (CBS) has not yet been implemented to its full potential yet it is one of the recommended methods for second generation HIV surveillance [1,2]. Second generation surveillance systems advanced beyond initial epidemic monitoring approaches that focused on aggregate numbers to use of individual-level clinical data. Within CBS, individual patient demographic attributes can be linked to key clinical events over time allowing for individual tracking. Hence, HIV cases are tracked from (1) diagnosis, (2) linkage to care, (3) antiretroviral treatment (ART), (4) viral suppression, and (5) other outcomes such as retention in care, transfer-out, and loss to follow up or death. This level of follow up is useful for developing epidemiological profiles at the smallest geographical units [3], monitoring of the HIV care and treatment clinical cascades, and measuring achievement of the Joint United Nations Program on HIV and AIDS (UNAIDS) Fast-Track 90-90-90 targets [4].

Case-based surveillance has advantages over aggregate data reporting systems since it uses individual-level data, allowing for better tracking of treatment course and outcomes. Case-based surveillance can also more accurately show trends and event sequences in the HIV epidemic, for example, trends of time to linkage to treatment from HIV testing or even changes in the clinical cascade over time [5]. Though CBS has been shown to be feasible in low resource settings [6], accuracy in CBS is contingent upon unique patient identification and correct record linkage from HIV diagnosis through the treatment course, due to the longitudinal nature of HIV care and multiplicity of data sources and care settings. Moreover, record linkage is useful for attaching records to a residence and geographic locality for example, in demographic and health surveillance systems where individuals are tracked routinely in their households [7], for data aggregation, and to facilitate correct assessment of program coverage.

There are 2 broad approaches to matching and records linking by using personally identifiable information (demographic data matching) and using a universal health care identifier (UHID) assigned to uniquely identify persons within a health care setting. Some of the earlier use cases for persons matching include immunization programs [8,9], and in other settings where unique identification is important such as a national census [10,11]. Though less common in settings such as HIV care and treatment programs, unique patient identification has recently and increasingly become important as patient volume grows in these settings. In HIV care and treatment, patient volume continually increases and so does the need for electronic medical records (EMRs). There are commensurate benefits of EMRs over paper records such as improved patient care coordination and clinical decision support [12]. Electronic medical records improve the capture of patient identifiers including UHIDs needed for longitudinal patient follow up. The utility of UHID for longitudinal follow up of patients has been demonstrated through correcting misclassification of the final patient outcomes such as loss to follow up in highly mobile populations. For example, in South Africa, a study among postpartum women found that a third may be misclassified as having been lost to care [13]. As a chronic condition, HIV care entails the use of HIV services by patients at multiple locations over a lifetime. Additionally, individuals may get an HIV diagnosis at one facility and choose to engage in HIV care at another location, they may receive a diagnosis in more than one care setting, and patients may move HIV care locations with or without notifying health care staff.

While UNAIDS recommends patient-centered colocation and integration of services across care settings such as antenatal care, tuberculosis, and HIV [4], colocation is not always feasible and hence tracking patients across the cascade of treatment can be difficult without a UHID and reliable EMR. Even when a government identification document is issued at adulthood, use of its unique number for reproductive and health care services is limited by acceptance and excludes younger populations. Additionally, name and location matching may be used where patient details such as names and locator information exist [14], but have limited utility in mobile populations. In the absence of a UHID, biometrics such as fingerprints are recommended [15] and may be used among HIV infected patients receiving care [16]. Other forms of patient identification, for example, the HIV comprehensive care clinic (CCC) medical record number used in Kenya suffers from low portability since they may not be permanent when a patient reinitiates care in a different facility. Program-identifiers have limited potential for a national surveillance system since they are unique to issuing facility. Hence, patients may acquire a new identifier when they transfer to a different facility resulting in unlinked data [17].

Given the chronic nature of HIV infection, integrating care across multiple service providers is essential. Nonetheless, unique patient identification in HIV programs, especially in Sub-Saharan Africa is rarely harmonized across service providers [18]. Without a unique patient identifier, if name and location data are available, they may be used to link medical records [14]. Therefore, demographic data have utility in records linkage. There are 2 types of algorithms for records matching, allowing for subsequent linkage and deduplication. The first is deterministic matching—a stepwise procedure in which sets of rules are used to pair up records based on actual or pseudo-identifiers identifying them as either a match or belonging to different persons. The second is score-based matching which refers to arithmetical models used to classify record pairs based on calculating a string distance measure quantifying how dissimilar 2 strings or words are to 1 another and applying a decision rule such as a score. The score is then used to determine whether duplicate records belong to the same individual.

Persons matching using score-based demographic data matching algorithms may be feasible for patient clinical encounter data and surveillance where demographic data is documented. However, there is a lack of data on the utility of score-based demographic data matching methods and how they compare with deterministic matching in low-resource settings including Sub-Saharan Africa. We used data from a pilot of HIV case-based surveillance in Siaya and Kisumu—two high HIV-burden counties in western Kenya to (1) compare deterministic and score-based patient matching algorithms and (2) propose an efficient algorithm for deduplicating and uniquely identifying HIV cases in CBS data collection and reporting in Kenya and similar settings.

## Methods

### Study Setting

This HIV case-based surveillance pilot was conducted between July 2015 and December 2015 in 124 facilities in Kisumu and Siaya counties. The facilities were selected to represent a variety of settings such as levels of care (dispensary, health center, subcounty, and county referral), use of an EMR versus paper records, and size of the patient population. Data were collected retrospectively to allow for at least four months of follow-up time from initial diagnosis, entry into care, or ART initiation within the study period. Data were collected by subcounty AIDS and sexually transmitted infections (STI) coordinators and Kenya Medical Research Institute (KEMRI) surveillance officers, and in some cases, facility staff. Data were entered from paper medical records and registers into the customized data entry platform for cases newly diagnosed or newly enrolled in HIV care from January through June 2015 using Android-based tablets and a standardized HIV case report form. Surveillance officers were trained in data collection using tablets and provided with login credentials. All surveillance officers signed a data confidentiality statement. As collected data contained patient names and other patient identifiers they were encrypted before transmission via a dedicated virtual private network in real-time to a server hosted on the Amazon cloud computing service. The staff at the National AIDS and STI Control Program (NASCOP) managed the data [19].

A case was defined minimally to include the date of diagnosis, age at diagnosis, gender, first name, and surname. Cases originated from the following 3 scenarios and analytical frameworks relating to the HIV care cascade. The first scenario is within HIV testing services (HTS). This scenario accommodates cases found within the same facility (cases that were tested at the facility and retested at the same facility hence having different dates of diagnosis). It also included cases that moved to a different facility (cases that tested at one facility and retested at a different facility). The second scenario is HTS-care. This accommodated HTS-to-care scenario in which cases were tested and linked within the same facility. It also included HTS-to-care cases that would be tested at one facility and then linked to care in a different facility. These 2 scenarios accounted for movement of persons diagnosed with HIV and accessing care within the same facility and clients that may test at one facility and access care in a different facility. The third scenario is within care scenarios. This included referrals and linkages from one facility to another. Similar to HTS-to-care linkage scenarios some cases had enrolled into care in one facility and throughout care transferred to another facility. However, HTS was not a source of data for the diagnosis information, and hence we did not have any testing location information for these cases.

### Data Collection

**Figure 1:**
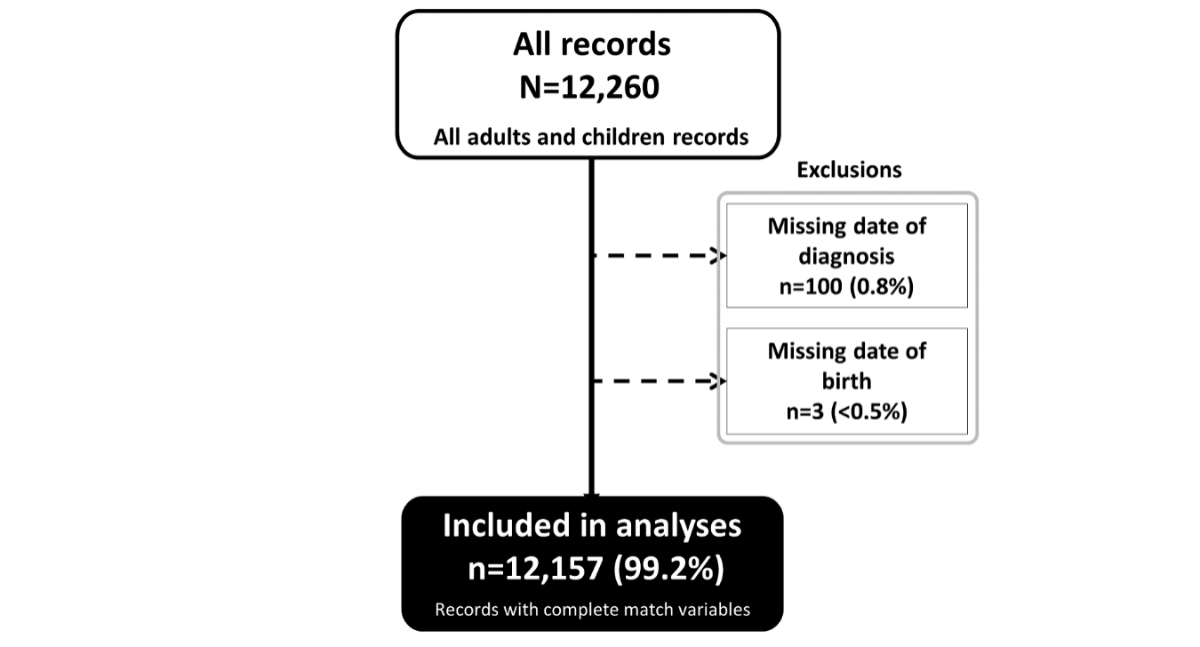
Number of records used for deterministic and probabilistic matching, HIV case-based surveillance in Kenya (2015).

Methods for data collection are described in the HIV case-based surveillance pilot report [19]. Briefly, data were extracted prospectively for everyone newly diagnosed or enrolled in care in a given 6-month period in the participating facilities and subsequent updating of sentinel events for those individuals. At the end of the pilot, we had 12,260 records but excluded 100 which had a missing date of diagnosis and 3 which had a missing date of birth before matching (Figure 1).

### Data Preparation and Standardization

We created analytical groups—also called “blocking” according to the scenarios described in the study setting before carrying out matching analyses to allow for comparability and faster processing,

Before carrying out matching processes, we standardized patient identifying fields used in matching. First, all blank spaces, commas, apostrophes, and dashes were stripped from first names middle names and surnames. Second, all string fields were converted to lower case. A Soundex [20] was created for the first names in all records since the first names are mostly of English origin. Third, we created double metaphone for middle names and surnames. Fourth, the year of birth was standardized to a four-digit number.

A potential patient identifier for the deduplication process is CCC number which is a unique patient number assigned at first clinical encounter once an HIV-infected patient has gone through triage and is ready for enrolment into a facility-managed HIV program. The CCC number is an 11-character code comprising a 5-digit unique facility code followed by a separator and a 5-digit sequentially facility-assigned unique number. We standardized CCC numbers to consider variations in recording (eg, use of spaces, slashes, dashes, adding leading zeros, and commas).

### Deterministic Matching

We used the following fields in deterministic matching (1) the first name, (2) surname, (3) gender, and (4) year of birth. To reduce mismatching due to variation in spellings of English first names, we used Soundex. We then created a “pseudo-unique key” combining the resulting Soundex values as well as gender, surname, and year of birth. The CCC numbers were used to match care records that were missed by using the “pseudo-unique key.”

### Score-Based Matching

**Figure 2:**
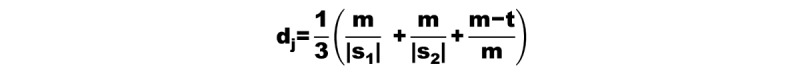
The Jaro-Winkler equation.

We separated the data according to the “blocking” scenarios described in the deterministic process. These blocking scenarios are necessary so that comparisons are made among potentially related records. We used R (an open-source software) in our study since it provided programming flexibility to implement the matching string preparation and matching process. We created a matching key field by including the data elements (1) first character of gender at birth, (2) Soundex of the first name, (3) secondary double metaphone of middle name, (4) secondary double metaphone of surname, and (5) year of birth. This produced strings such as “fF465aknannk1983,” “fI650aknkannk1990” (where middle name secondary double metaphone was available), and “fG620ans1994” (where secondary double metaphone of the middle name was unavailable). We then implemented Jaro and Jaro-Winkler string matching and Levenshtein and Damerau-Levenshtein string edit distance algorithms in the R stringdist package [21,22]. String score-based matching was conducted using ratios of matching strings, and a penalty was applied for the first 4 characters when Jaro-Winkler algorithm is used as in the formula (Figure 2) where *d*_j_ is the Jaro-Winkler distance score, *m* is the number of matching characters, |*s*_1_| is length of string 1, |*s*_2_| is length of string 2 and *t* is half the total transpositions or the number of matching (but different sequence order) characters divided by 2. String edit distance calculations, on the other hand, quantify how different 2 strings or words are to one another by counting the minimum number of deletions, insertions, substitutions and transposition operations required to transform 1 string into the other. Score-based methodologies are based on the Fellegi-Sunter linkage rule that classifies a record pair as matching or nonmatching [11]. The score level to determine a match is determined a priori or based on experience by the user and dependent on the setting. For our case, a score of 98% and above was considered sufficient to determine a match. When we implemented the Jaro and Jaro-Winkler methods, we set a standard penalty factor to 0.1. This penalizes matches based on similarity at the beginning of the string to give favorable ratings to strings that match from the beginning for a set prefix length of up to 4 characters according to Winkler and Cohen [11,23]. The penalty factor is added to discount matches that are found based on up to a maximum of first 4 characters since in string writing, the person recording is more likely to make an error after the first 4 characters. We considered the 4 weights (ω) applicable to the Levenshtein and Damerau-Levenshtein methods (1) deletion (ω=0.8), (2) insertion (ω=0.8), (3) substitutions (ω=1), and (4) transposition (ω=0.5). For the Levenshtein method, the penalty for substitution is ignored [22].

Due to possibilities of age variations for the same person accessing HTS and care services at differing periods, the numeric comparator age, with a variation of not more than 12 months within identified matches was considered sufficiently close for confirming a match. We compared deterministic and score-based processes for unique case identification regarding the number of matches yielded and the deduplication extent achieved within the scenarios. We also assessed match yield when HTS and HTS-care records were treated as mutually exclusive versus as a combined set. Regardless of approach, total yield was a sum of duplicates from all scenarios.

### Postmatch Processing

Based on the date of HIV diagnosis, we carried out extra steps to determine how to retain unique records after the matching process. If the retained and duplicate records had conflicting dates of diagnosis, we retained the records with the earliest date of diagnosis. For retained records, we maximized completeness of data for all fields by comparing with the duplicate records. Whenever a retained record had missing data that was in duplicate record, an append merge was carried out to overwrite missing values with the nonmissing value from the matched record.

### Ethical Considerations

Ethical approval was obtained from the KEMRI (SSC #2827) and the Office of the Associate Director for Science, Centers for Disease Control and Prevention (CDC) with tracking #2014-136. Access to data used in these analyses was password protected, and all study coordinators, data abstractors, and analysts signed a confidentiality form.

## Results

### HIV Case Records and Demographic Data Variables

A total of 12,260 records were collected. We excluded 100 (0.8%) records due to missing dates of diagnosis, and 3 (0%) missing the date of birth (Figure 1). The final data set used for the matching exercise included 12,157 records representing adult and pediatric cases. From these records and before data deduplication, 33.5% (4073/12,157), 9.0% (1091/12,157), and 57.5% (6,993/12,157) corresponded to HTS, HTS-care and within care scenarios respectively. In Table 1, completeness and uniqueness of variables used to construct score-based matching string are presented. In the entire data set, gender, year of birth, first name and surname were 100% complete while the middle name was missing for 38% of the records. First names were less unique than surnames: 8.2% (1002/12,157) versus 19.1% (2321/12,157). When Soundex was applied to standardize the English first names, 273/12,157 (2.2%) remained unique compared to 1002/12,157 (8.2%) of the original unstandardized format. When secondary double metaphone was applied to standardize the middle and surnames, 2.6% (316/8772) and 3.1% (373/12,157) respectively remained unique compared to 13.1% (1150/8,772) and 19.1% (2321/12,157) of the original unstandardized format. The similarity of names varied by setting (Table 1).

### Matches Identified

Out of the 12,260 records, 12,157 (99.2%) were used in the analyses. Using the deterministic method, 67/12,157 (1.6%) records were matches in HTS, 164/12,157 (15.0%) in HTS-care, and 204/12,157 (2.9%) in the care-only scenario. This yielded a total of 435/12,157 (3.6%) matches and 11,722 unique cases across the testing and, care and treatment cascade (Table 2).

Overall, of the score-based methods, Jaro-Winkler yielded the most duplicate records (686/12,157, 5.6%), Jaro yielded the fewest (546/12,157, 4.5%), and both Levenshtein and Damerau-Levenshtein yielded the same number (563/12,157, 4.6%). Specifically, duplicate records yielded by method were: (1) Jaro 5.7% (234/4073) within HTS, 0.4% (4/1,091) in HTS-care, and 4.4% (308/6993) within care, (2) Jaro-Winkler 7.4% (302/4073) within HTS, 0.5% (6/1091) in HTS-care, and 5.4% (378/6993) within care, (3) Levenshtein 6.4% (262/4073) within HTS, 0.4% (4/1091) in HTS-care, 4.2% (297/6993) within care, and (4) Damerau-Levenshtein 6.4% (262/4073) within HTS, 0.4% (4/1091) in HTS-care, and 4.2% (297/6993) within care.

**Table 1:** Completeness and uniqueness of demographic fields used in the matching process for HIV case-based surveillance in Kenya 2015 (N=12,157).

Fields	Completeness (%)	Unique^a^ (n)	Out of n (%)
Gender^b^	100	2	12,157 (0)
Year of birth	100	6	12,157 (0)
First name	100	1002	12,157 (8.2)
Soundex of first name	100	273	12,157 (2.2)
Middle name	72	1150	8772 (13.1)
Phonetic middle name^c^	72	316	8772 (3.6)
Surname	100	2321	12,157 (19.1)
Phonetic surname^c^	100	373	12,157 (3.1)

^a^Unique refers to similar occurrences of the field (eg, only two types of gender).

^b^Two statuses possible (male or female).

^c^Secondary double metaphones for standardizing Kenyan native names.

**Table 2:** Scenarios in HIV diagnosis, care and treatment cascade, and deduplication yield for HIV case-based surveillance in Kenya 2015.

Scenarios	Deterministic matching method, n (%)	Matches identified for each score-based matching algorithm, n (%)			
		Jaro	Jaro-Winkler	Levenshtein	Damerau-Levenshtein
All^a^ (N=12,157)	435 (3.6)	546 (4.5)	686 (5.6)	563 (4.6)	563 (4.6)
HTS^b^ (n=4037)	67 (1.6)	234 (5.7)	302 (7.4)	262 (6.4)	262 (6.4)
HTS-care^c^ (n=1091)	164 (15.0)	4 (0.4)	6 (0.5)	4 (0.4)	4 (0.4)
Care only^d^ (n=6993)	204 (2.9)	308 (4.4)	378 (5.4)	297 (4.2)	297 (4.2)
Unique^e^	11,722 (96.4)	11,611 (95.5)	11,471 (94.4)	11,594 (95.4)	11,594 (95.4)

^a^Summed up for all the scenarios.

^b^HTS: HIV testing services (records where data were primarily from the HTS setting and the records contained HIV diagnosis data only).

^c^HTS-care (records that contained both HTS and HIV care information).

^d^Care only (records from primarily HIV care with no additional HTS records).

^e^Unique records after deduplication.

### Jaro-Winkler Yield for Mutually Exclusive and Combined Data Sets

**Figure 3:**
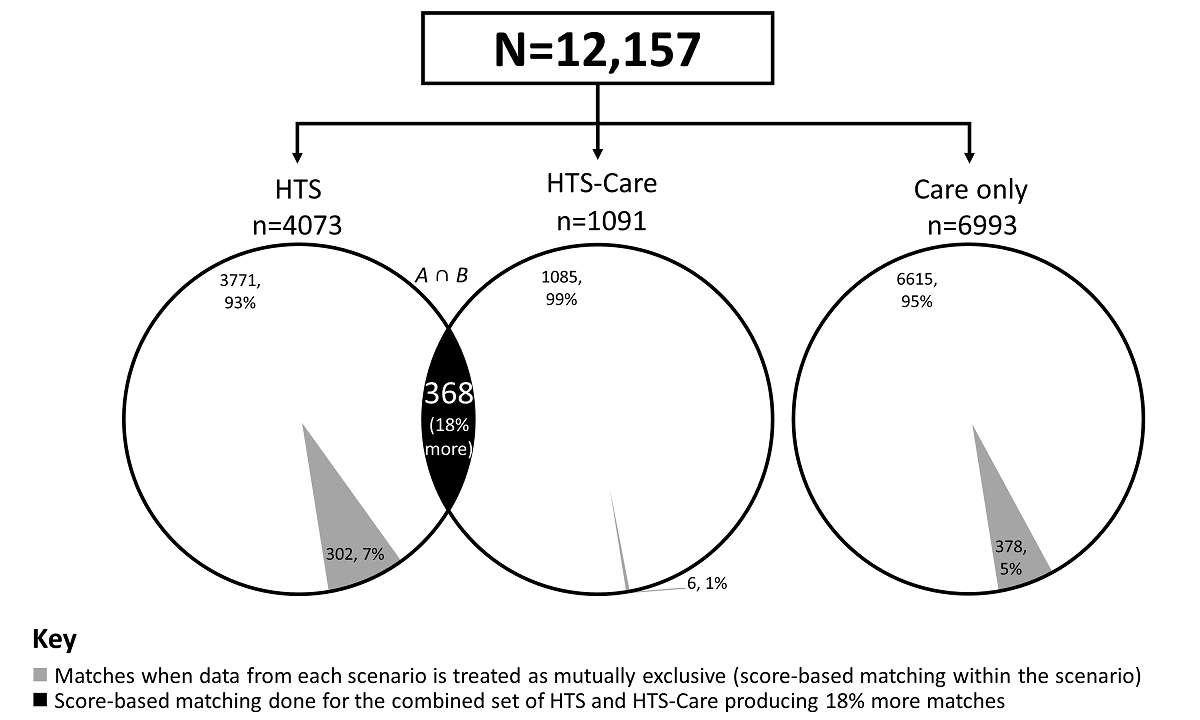
Percent match yield by blocking scenarios using Jaro-Winkler score-based matching, HIV case-based surveillance in Kenya (2015). HTS: HIV testing services; HTS-Care: records from HTS-care scenarios; Care only: records from care scenarios only. A ∩ B indicates that the intersection of HTS and HTS-case records yields 386 matches (18% more matches than in mutually exclusive matching).

A comparison of Jaro-Winkler yield for mutually exclusive and data sets that were combined across the scenarios is presented in Figure 3. When scenarios were treated as mutually exclusive, Jaro-Winkler score-based matching algorithm yielded 7.0% (302/4073) matches in the HTS scenario, 1% (6/4073) in the HTS-care scenario compared to a higher yield of 7.1 % (368/5164) when the 2 scenarios were treated as 1 block.

### Steps for Score-Based Matching and Considerations

**Figure 4:**
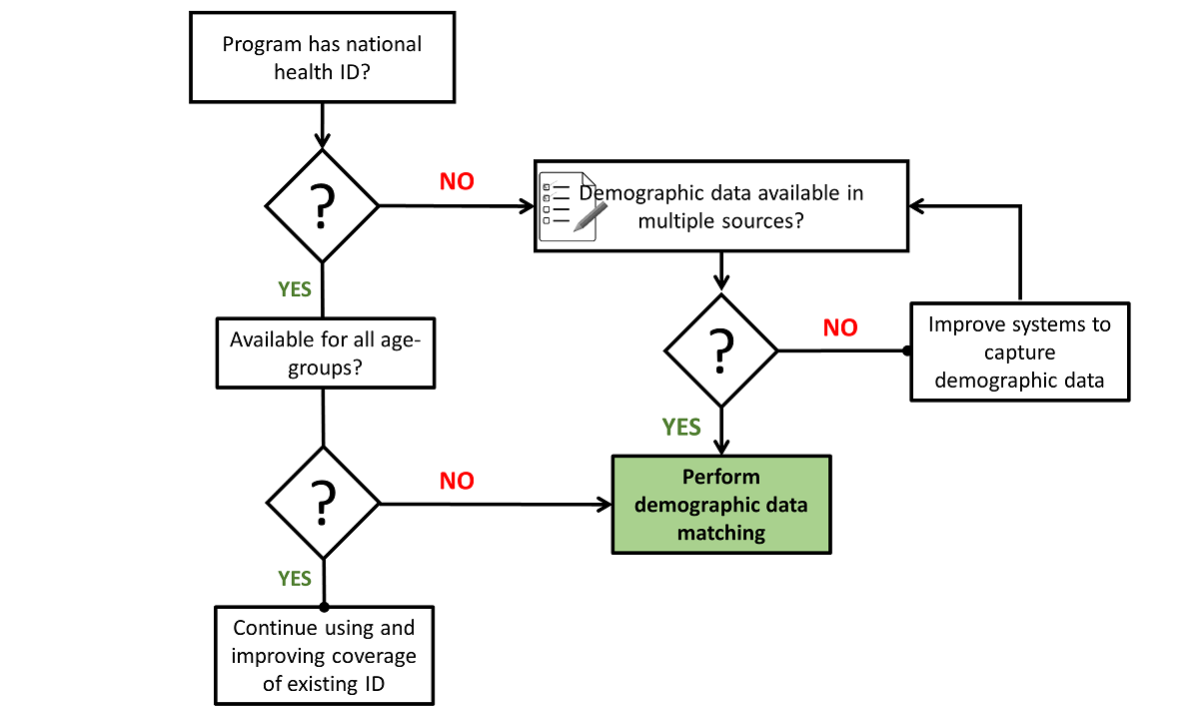
Decision model for when to use score-based matching.

Based on the outcomes of the score-based matching process, we propose a procedure comprising of 7-steps that is easy to apply to quickly match and link unique cases across HIV care settings (Textbox 1). To decide whether or not to use demographic data matching, we propose a decision model (Figure 4).

**Expandable simplified steps used in the demographic data matching process.**

**Step 1: Select data sources**

- Select data sources with common fields
- If additional sources are available, add to the list

**Step 2: Prepare the data**

- Cleaning and coding
- Standardizing fields

**Step 3: Create a match-string**

- Ensure mutually exclusive blocks
- Test internal validity

**Step 4: Create blocks**

- Ensure mutually exclusive blocks
- Test internal validity

**Step 5: Run the matching algorithm**

- Apply a match rate of ≥98%
- Test in a small identified data set and adjust the match rate

**Step 6: Merge the data**

- Update records that need an update
- Create a master patient index

**Step 7: Adjudication, quality checks, and use cases**

- For care coordination, recheck that the matches are correct
- For surveillance and indicator reporting, use a combination of the matched but deduplicated records and the unmatched records

## Discussion

### Principal Findings

Universal health care identifiers are recommended and ideal for patient-centered monitoring and CBS [24,25]. However, in low resource settings, their use is limited. In the interim, demographic data score-based matching algorithms can play an important role in improving the quality of CBS data as well as patient-centered care. We have demonstrated that score-based methods succeeded in patients matching and identifying more matches compared to the deterministic process. It is possible to match cases, merge sentinel events, and enhance the completeness of individual deduplicated data using this process. Consequently, this improves accuracy in CBS and other longitudinal encounter data. The process also has a dual utility of allowing better care coordination and patient management at the facility level and improved HIV surveillance at a higher subnational or national level. The matching process can be inbuilt in EMRs and at patient registries to allow for lookup of already registered patients at the facility level. This may improve processes, patient flow and avoid unnecessary double entry. We also demonstrate that we can do enough matching in the absence of a UHID to move ahead with CBS implementation in low-resource settings such as Kenya. As such, lack of a UHID should not stifle movement towards the use of CBS.

### Score-Based Matching Yield

Our study compared 4 variants of score-based string-distance matching methods. The Jaro-Winkler distance method was found to perform better in score-based matching since it gave the best yield while considering common spelling mistakes and logical combination of demographic fields. In developed countries, it has been shown that about 5% to 10% of medical records may be duplicate [26], which compares well with our results. Jaro-Winkler has been proposed as a method over other string-matching algorithms since it was designed with relatively short strings in mind [21], hence may be best suited to our setting. In addition, it works well when the name beginnings are the same [27]. For that reason, we standardized beginning of the match strings by using a Soundex of the English names and using secondary double metaphone of middle and surnames. Further, a decision was made to add the first character of gender at birth to the beginning of the string to improve the accuracy of the matching score.

### Application Considerations

Although we used R in our analyses and matching process, open source software such as CDC Registry Plus Linkplus [28], which was originally developed for cancer registries has been explored in low-resource HIV care settings for example in Haiti [29]. Other Web-based applications that have utility for fuzzy matching and record cleaning, for example, Freely Extensible Biomedical Record Linkage [6], may have potential. However, post-match processing is necessary to achieve a high degree of true matches. A certain degree of human adjudication may be necessary especially when testing the algorithms. Users of off-the-shelf solutions such as Linkplus should take caution since many mismatches may be likely to be true matches [29]. The use of current English name-based Soundex algorithms is not appropriate for Kenyan names. In creating unique identifiers that contain a Soundex component, variations of the first name can yield a different Soundex since the first character is always part of the Soundex [20]. A visual inspection of matches based solely on Soundex of first and surname showed a high false-positive rate. Research on how to construct a Soundex algorithm for Kenyan names may be useful as has been successfully done in Japan, India, and South Africa [30-32]. We determined that using a double metaphone had discriminatory power for Kenyan names and hence we used it for middle and surnames.

### Limitations

Our study has several limitations. First, the choice of a combination of several fields for a concatenated “pseudo-unique key” may not be optimal. However, we developed the matching string taking advantage of existing identifiers in our data. First names in Kenya are usually English baptismal or anglicized names. We took advantage of this to standardize names that are misspelled using Soundex. Other challenges include manual transcription errors during patient transfers and assigning of new numbers for transfer-in patients. Despite these limitations, we were able to merge the cases based on the names, gender, date of birth and CCC number in the within care scenario and hence identify potential matches in the deterministic process. Finally, many studies have applied common measures of validity such as positive predictive value, sensitivity, and specificity [33]. Unlike those studies, we did not have a gold standard for comparison in the pilot.

The choice of which string distance score-based algorithm to use largely depends on the nature of the match strings and the nature of typographic errors [21]. Choice of the matching string is therefore important. For example, deterministic matching yielded more duplicates for the HTS to care scenario (15%) compared to 4.6% to 7.1% across the score-based methods. This may be because a rigorous manual assessment of possible matches was done using the CCC numbers such that matches within the HTS to care scenario were more efficiently captured. Minimalistic demographic fields were used in score-based matching across all scenarios, and the CCC number was not included in the process.

### Conclusions and Recommendations

There has been an ongoing discussion and suggested approaches for countries to consider in developing UHIDs [17,34]. If, and when implemented, UHIDs would have the highest potential to mitigate challenges with a unique identification and record linkage for an expanded national CBS system. This benefit extends to other health sectors as countries move towards universal health care. The recent World Health Organization guidelines for patient-centered monitoring advocate for using unique patient identifiers instead of names [25]. However, where there is no UHID, a unique patients’ deduplication algorithm based on available demographic data is necessary and feasible. Such an algorithm would improve monitoring of the HIV epidemic including the UNAIDS Fast-Track 90-90-90 targets.

We propose a stepwise process that builds up from first identifying data sources and blocking scenarios. This should be followed by an examination of the data quality using completeness as a measure coupled with quality improvement measures through routine data quality audits. The next step involves developing a matching key, lower-level deduplication and finally cross-examination, validation and sending of CBS data to the national level for surveillance. Although validation of the score-based approach is a necessary extra step, this may be best done with data sets from settings where a gold standard is available such as those utilizing biometric finger vein technologies for patient identification. Given that these settings are rare, we suggest that programs identify a percentage that best suits their setting and resources for validation purposes. A decision model such as the one presented in Figure 4 may help programs to decide whether or not to use demographic data matching. Comparing score-based matches to gold standard data in Kenya and similar settings offer an opportunity for future work in search of alternatives for patient matching. In the meantime, score-based demographic data matching has utility for improving the quality of data in monitoring the 90-90-90 cascade and in other health care settings where patients are longitudinally followed.

## Abbreviations

ART: antiretroviral treatment
CBS: case-based surveillance
CCC: comprehensive care clinic
CDC: Centers for Disease Control and Prevention
EMR: electronic medical record
HTS: HIV testing services
KEMRI: Kenya Medical Research Institute
NASCOP: National AIDS and STI Control Program
STI: sexually transmitted infection
UHID: universal health care identifier
UNAIDS: Joint United Nations Program on HIV and AIDS

## References

[1] (1999). Guidelines for national human immunodeficiency virus case surveillance, including monitoring for human immunodeficiency virus infection and acquired immunodeficiency syndrome.

[2] UNAIDS/WHO Working Group on Global HIV/AIDSSTI Surveillance (2011). Guidelines for Second Generation HIV Surveillance-an update: Know your epidemic.

[3] (2015). Joint United Nations Programme on HIV/AIDS (UNAIDS).

[4] Joint United Nations Programme on HIV/AIDS (UNAIDS).

[5] Rehle T, Lazzari S, Dallabetta G, Asamoah-Odei E (2004). Second-generation HIV surveillance: better data for decision-making. Bull World Health Organ.

[6] Harklerode R, Schwarcz S, Hargreaves J, Boulle A, Todd J, Xueref S, Rice B (2017). Feasibility of Establishing HIV Case-Based Surveillance to Measure Progress Along the Health Sector Cascade: Situational Assessments in Tanzania, South Africa, and Kenya. JMIR Public Health Surveill.

[7] Christen P, Churches T (2005). A probabilistic deduplication, record linkage and geocoding system.

[8] (2013). National Center for Immunization and Respiratory Disease (NCIRD).

[9] Angeloni M (2004). Probabilistic Record Matching and Deduplication Using Open Source Software.

[10] Jaro MA (1989). Advances in Record-Linkage Methodology as Applied to Matching the 1985 Census of Tampa, Florida. Journal of the American Statistical Association.

[11] William EW, Thibaudeau Y Research Report.

[12] Oluoch T, Katana A, Ssempijja V, Kwaro D, Langat P, Kimanga D, Okeyo N, Abu-Hanna A, de KN (2014). Electronic medical record systems are associated with appropriate placement of HIV patients on antiretroviral therapy in rural health facilities in Kenya: a retrospective pre-post study. J Am Med Inform Assoc.

[13] Clouse K, Vermund SH, Maskew M, Lurie MN, MacLeod W, Malete G, Carmona S, Sherman G, Fox MP (2017). Mobility and Clinic Switching Among Postpartum Women Considered Lost to HIV Care in South Africa. J Acquir Immune Defic Syndr.

[14] Dusetzina SB, Tyree S, Meyer A-M, Meyer A, Green L, Carpenter WR (2014). Linking Data for Health Services Research: A Frame work and Instructional Guide.

[15] Kabudula CW, Clark BD, Gómez-Olivé FX, Tollman S, Menken J, Reniers G (2014). The promise of record linkage for assessing the uptake of health services in resource constrained settings: a pilot study from South Africa. BMC Med Res Methodol.

[16] Otieno J The Star, Kenya Sep.

[17] Beck EJ, Shields JM, Tanna G, Henning G, de Vega I, Andrews G, Boucher P, Benting L, Garcia-Calleja JM, Cutler J, Ewing W, Kijsanayotin B, Kujinga T, Mahy M, Makofane K, Marsh K, Nacheeva C, Rangana N, Vega MFR, Sabin K, Varetska O, Macharia Wanyee S, Watiti S, Williams B, Zhao J, Nunez C, Ghys P, Low-Beer D (2018). Developing and implementing national health identifiers in resource limited countries: why, what, who, when and how?. Glob Health Action.

[18] World Health Organization (2007). IMAI and IMCI tools.

[19] National AIDS and STI Control Programme (NASCOP) (2016). Case Based Surveillance of HIV in Kenya: Results of a Pilot Conducted in Kisumu and Siaya Counties, 2015. Case Based Surveillance of HIV in Kenya.

[20] (2007). The US National Archives.

[21] van der Loo M The R Journal.

[22] van der Loo M, van der Laan J, Logan N, Muir C, R Core Team (2018). https://cran.r-project.org/web/packages/stringdist/stringdist.pdf.

[23] Cohen W, Fienberg S, Ravikumar P, Fienberg S (2003). Proceedings of IJCAI-03 Workshop on Information Integration on the Web.

[24] World Health Organization (2017). Adapting and Implementing New Recommendations on HIV Case surveillance.

[25] (2017). Consolidated Guidelines on Person-Centred HIV Patient Monitoring and Case Surveillance.

[26] Fox Leslie Ann, Sheridan Patty Thierry (2004). Advance healthcare network.

[27] Christen P (2006). A Comparison of Personal Name Matching: Techniques and Practical Issues. Data Mining Workshops.

[28] CDC (2007). National Program of Cancer Registries (NPCR).

[29] Chris D, Puttkammer N, Arnoux R, Kesner F, Griswold M, Zaidi I, Anthony Y, Joseph P, Marston B (2016). Validating Procedures used to Identify Duplicate Reports in Haiti's National HIV/AIDS Case Surveillance System. J Registry Manag.

[30] Baruah D, Kakoti Mahanta A (2015). Design and Development of Soundex for Assamese Language. IJCA.

[31] Shah R, Kumar Singh D (2014). Improvement of Soundex Algorithm for Indian Language Based on Phonetic Matching. Int J Comput Sci Eng Appl.

[32] Ndyalivana Z (2017). Development of Soundex Algorithm for IsiXhosa Language.

[33] Pinto da Silveira D, Artmann E (2009). Acurácia em métodos de relacionamento probabilístico de bases de dados em saúde: revisão sistemática. Rev Saúde Pública.

[34] (2014). Joint United Nations Programme on HIV/AIDS (UNAIDS).

